# High Fetuin-A Levels in Children with Celiac Disease

**DOI:** 10.5152/eurasianjmed.2022.21293

**Published:** 2022-06-01

**Authors:** Nezahat Kurt, Fatma Betul Ozgeris, Burcu Volkan, Mehmet Ali Gul, Atilla Cayir

**Affiliations:** 1 Departman of Medical Biochemistry, Faculty of Medicine, Erzincan Binali Yildirim University, Turkey; 2 Departman of Nutrition and Dietetics, Faculty of Healthy Sciences, Ataturk University, Turkey; 3 Department of Pediatric Gastroenterology, Hepatology and Nutrition, Marmara University School of Medicine, Istanbul, Turkey; 4 Departman of Medical Biochemistry, Faculty of Medicine, Amasya University, Turkey; 5 Department of Pediatric Endocrinology, Erzurum Regional Training and Research Hospital, Erzurum, Turkey

**Keywords:** Celiac disease, Fetuin-A, bone mineral density

## Abstract

**Objective:** Fetuin-A is a multifunctional non-collagen protein that plays a role in bone mineralization. Celiac disease is a chronic inflammatory disorder of the small intestine due to exposure to gluten. In this research, it was aimed to investigate levels of Fetuin-A and its relationship with bone mineral density in children with celiac disease.

**Materials and Methods:** The study was conducted on 59 children with celiac and 29 healthy children. The celiac disease group was composed of three groups, newly diagnosed, gluten-free diet compliant and, non- gluten-free diet compliant patients. Serum Fetuin-A concentrations were measured by an enzyme-linked immunosorbent assay kit. Measurement of bone mineral density was performed a dual-energy x-ray absorptiometry.

**Results:** Serum Fetuin-A levels were 136.85 ± 38.09 µg/L and 112.95 ± 44.39 µg/L in the celiac disease and healthy control groups, respectively. There was a statistically significant difference between groups in levels of serum Fetuin-A (*P* < .05). A significant positive correlation was observed between serum Fetuin-A and bone mineral density Z-score in the celiac patients.

**Conclusion:** Increased Fetuin-A levels and positive correlation between Fetuin-A and bone mineral density in children with celiac disease suggest that Fetuin-A may be a biomarker for celiac disease.

**Main Points**

- Fetuin-A levels are higher in children with celiac.
- Fetuin-A may play a role in osteopenia.
- Fetuin-A has a positive correlation with bone mineral density.

## Introduction

Celiac disease (CD) is an autoimmune enteropathy triggered by ingested gluten peptides, develops in genetically susceptible individuals.^1^ Clinical manifestations of CD can be ranged the gastrointestinal symptoms such as malabsorption, diarrhea, vomiting, weight loss, and abdominal discomfort, to extraintestinal symptoms, including osteoporosis, anemia, dermatitis herpetiformis, infertility, and neurological problems. Extraintestinal manifestations may be the first symptoms of the disease, especially in later ages and adults, without gastrointestinal symptoms.^2^ Celiac disease may result in impaired bone mass and mineral metabolism and metabolic osteopathy.^3^ Intestinal malabsorption and inflammation contribute to the pathophysiology of bone damage in CD. Many studies have been reported low bone mineral density (BMD) in CD.^4–6^

Fetuin-A (a-2 Heremans Schmid glycoprotein) is a multifunctional protein synthesized from the liver. It is found in high concentrations in serum.^7^ It makes up 25% of the non-collagen proteins in bone and is, therefore, one of the 2 most abundant non-collagen proteins.^8^ Bone Fetuin-A is probably synthesized hepatically and delivered to the bone via the bloodstream.^9^ Many studies have shown that Fetuin-A mediates mineral transfer in the general circulation and the extracellular area.^10–12^ It binds with a higher affinity to calcium phosphate and calcium carbonate and a lower affinity to magnesium phosphate.^13^ Price et al^14^ showed that Fetuin-A is necessary for determining the location of mineralization in their in vitro study. It has been observed that mineral accumulation in the presence of Fetuin-A is in collagen and outside of collagen in its absence.^14,15^ In a research, involving older men and women it was suggested that circulating Fetuin-A is associated with BMD, which is more pronounced in women. A similar finding was reported in another study among elderly women.^16,17^ There is no study that gives information about Fetuin-A levels, and the relationship between it and bone metabolism in children with CD. The present study aimed to investigate the Fetuin-A levels and the relationship between BMD and Fetuin-A in children with CD.

## Materials and Methods

### Ethics Committee

The approval for this research was obtained from the Clinical Research Ethics Committee of Atatürk University, Faculty of Medicine (B.30.2.ATA0.01.00/454). Informed consent was obtained from all individual participants included in the study.

### Patient and Control Group

The present study included 59 children with CD and 29 children without any health problems (HC). The diagnosis of CD was based on the criteria of the European Society for Pediatric Gastroenterology, Hepatology, and Nutrition.^18^ The CD group was divided into 3 groups: 18 previously diagnosed and compatible with diet (GDF), 20 previously diagnosed and incompatible with diet (Non-GDF), and 21 newly diagnosed (ND). According to anti-tissue transglutaminase (anti-tTG) IgA levels, it was defined as GFD (compliant with gluten-free diet) for 0-20 U/mL and Non-GFD (incompatible with the gluten-free diet) for >20 U/mL. The mean follow-up period of previously diagnosed patients was 2.6 years. Those who had type-1 diabetes mellitus, hyperthyroidism, and a systemic disease were excluded from the study in the CD group.

Height and weight were measured in CD and HC groups. Age-related body mass index Z-score (BMI-Z score) was calculated according to data from the general Turkish pediatric population.^19^

### Measurement of Bone Mineral Density

Dual-energy x-ray absorptiometry (g/cm^2^) was used to estimate the BMD for the CD group. The absolute BMD for each patient was expressed as a Z score when compared with the mean BMD of the age- and sex-matched healthy control group. For pediatric individuals, the definition of low bone mass was defined as a BMD Z score is ≤−2.0 adjusted for age, sex, and body size.^20^

### Biochemical Analysis

Blood samples taken from all participants were put into a serum tube and centrifuged at 1.000 × g for 15 minutes, and all serum specimens were stored at −80°C until the day of analysis. Serum calcium (Ca), phosphate (P), and magnesium (Mg) levels were assayed by the clinic autoanalyzer (SYCHRON LX; Beckman Coulter, Fullerton, CA, USA). Human anti-tTG IgA levels were detected by means of an immunoenzymatic method. Serum concentrations of Fetuin-A were measured using an enzyme-linked immunoassay (Fetuin A ELISA kit; ref number: DK0128, DiaMetra, Italy) and according to the manufacturer’s standard protocol. The serum samples and the ELISA kits were kept at room temperature for approximately 2 hours on the study day. Fetuin-A analysis was performed by a multiplate reader spectrophotometer (XS Powerwave, BioTEK, USA). Serum Fetuin-A levels were calculated as micrograms per liter.

### Statistical Analysis

Statistical analysis was performed using the statistical software program, Statistical Package for the Social Sciences, for Windows v.20 (IBM SPSS Corp.; Armonk, NY, USA). The variables were investigated using the Shapiro–Wilk test to determine normal distribution. Descriptive analyses were presented using mean and standard deviation (mean ± SD) or median, minimum, and maximum (med (min-max)). The differences in parametric and non-parametric data between CD and HC were calculated using Student’s *t* distribution and Mann–Whitney *U* test, respectively. Also, the Kruskal–Wallis test was used for statistical analysis of the celiac subgroups. The correlations were calculated using the Spearman rank test. Differences were regarded as significant at *P* < .05.

## Results

The demographic characteristics and biochemical findings of children with CD and the HC are shown in Table 1. The data of height, weight, and BMI Z-score in the CD group was significantly lower than the healthy control group (*P* < .001).

Serum Ca levels were not significantly different between the CD and HC (*P* > .05). While serum P values were significantly higher in the CD group (*P* < .001), serum Mg levels were significantly lower (*P* < .001).

**Figure 1.**
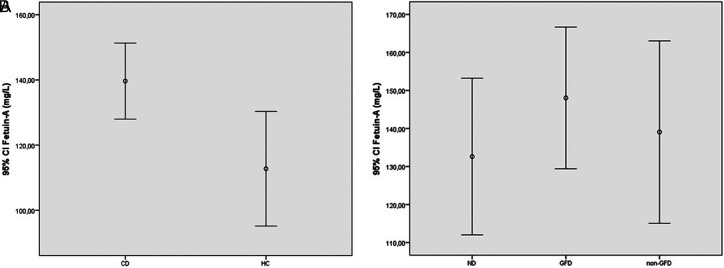
Serum Fetuin-A levels in patient and healthy control group (A), Serum Fetuin-A levels in celiac subgroups (B).

There was a statistically significant difference between HC and CD groups in terms of serum Fetuin A level (Table 2). Serum Fetuin-A levels were significantly higher in celiac patients (136.85 ± 38.09 mg/L) than the HC group (112.95 ± 44.39 mg/mL, *P* < .05), (Figure 1a).

In the CD subgroups, patients in the ND group had lower BMI than those in the Non-GFD and GFD groups (*P* < .05) (Table 1). Serum Ca values were significantly lower in ND patients, however, no significant difference was observed in serum Fetuin-A (Figure 1b), P, Mg, and BMD Z-scores among the three groups (Table 2).

**Figure 2.**
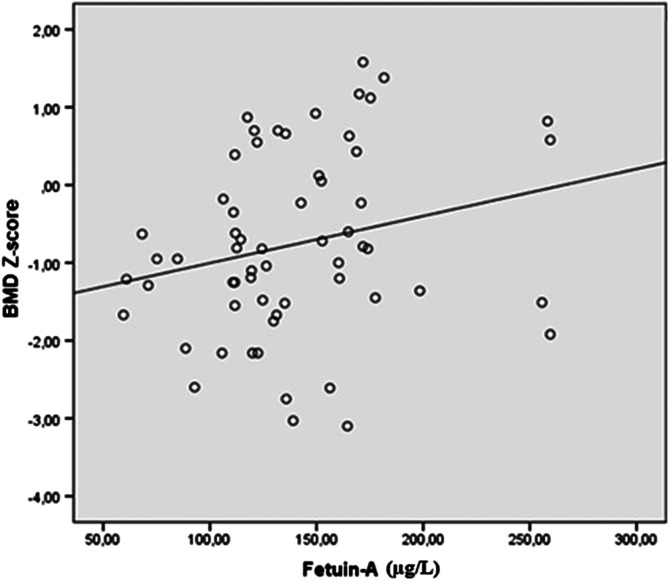
Correlation between Fetuin-A level and BMD Z-score in patient (*r = *0.257, *P* < .05).

As seen in Figure 2, in the patient group, a significant positive correlation was found between Fetuin-A levels and BMD (*r* = 0.257, *P* = .047).

## Discussion

This study investigated Fetuin A levels and the relationship of Fetuin-A to BMD in children with CD. Our results show that serum Fetuin-A concentrations are higher in patients with CD than in the healthy control group. Also, a positive correlation was observed between Fetuin-A and BMD in CD patients.

Many studies were shown that children with CD have lower bone mineral density than healthy children.^5,6,21,22^ In our research, we did not observe any difference among ND, good GFD, and non-GFD groups.

Fetuin-A is a liver-derived plasma protein. It is the most abundant globular plasma protein in fetuses and young children.^23^ Its best-known function is related to mineralization biology. It buffers mineral ion supersaturation by binding small clumps of calcium and phosphate that may form abnormally in circulation.^24,25^ Therefore, it acts as an important circulatory inhibitor of ectopic calcifications by moving these calciprotein particles to the organic matrix of the bone. Studies have shown that Fetuin-A is a serum protein that supports bone mineralization in vitro and in vivo.^16,26^

In research conducted with 508 healthy elderly people between the ages of 70-79 by Ix et al.^16^ they reported that high Fetuin-A levels were associated with high BMD in women. Rasul et al^27^ have observed an association of Fetuin-A levels with markers of bone turnover in patients with type 2 diabetes. In the first and only study on Fetuin-A levels in children, no difference was found in the Fetuin-A levels between children with cow’s milk allergy and healthy children.^28^ Ozkan et al.^29^ in their study on postmenopausal women, observed lower Fetuin-A levels in the osteoporosis group compared to the healthy group. They found a statistically insignificant positive correlation between Fetuin-A and lumbar and femoral BMD.

There are no studies on Fetuin-A in patients with celiac disease in the pediatric, adult, or elderly in the literature. The study we conducted is the first study on this subject, and the mean of Fetuin-A concentrations was found to be significantly higher in CD patients. However, there was no observed difference among CD subgroups. Decreased mineral absorption due to intestinal malabsorption causes low BMD in children with celiac disease. When the results of our study are evaluated, it may be thought that due to the role of Fetuin-A in bone mineralization, it is more synthesized in patients with celiac disease to provide bone mineralization at low mineral levels. Besides, finding a correlation between Fetuin-A and BMD in the group with CD supports this hypothesis. However, new research is needed to explain the mechanisms related to this.

In conclusion, increased Fetuin-A levels and a positive correlation between Fetuin-A and BMD in children with celiac disease suggest that Fetuin-A may a biomarker for celiac disease.

### Limitations of the Study

It is a limitation of this research that the number of cases (especially CD subgroups) is low. Participants who received treatment for celiac diseases such as vitamin D and calcium affecting bone metabolism could not be excluded.

## Figures and Tables

**Table 1.** Age, Height, Weight, and BMI-Z Score Data of the Study Groups

	CD Group Mean ± SD or Med (Min-Max)(N = 59)					HC Group Mean ± SD or Med (Min-Max) (N = 29)	*P*
	ND (N = 21)	GFD (N = 18)	Non-GFD (N = 20)	*P*	Total CD (N = 59)		
Age (year)	11.7 ± 3.3	11.5 ± 2.5	12.8 ± 2.4	.305	12.0 ± 2.8	13.0 ± 2.3	.104
Height (cm)	132.5 (104.1 to 155.2)	138.9 (108.2 to 166.4)	144.9 (112.1 to 160.1)^a^	.121	138.4 (112.1 to 166.1)^b^	150.4 (115.1 to 175)	.000
Weight (kg)	15.2 (12.4 to 21.2)	16.4 (11.9 to 30.2)^a^	17.4 (13.8 to 21.8)^a^	.373	16.1 (11.9 to 30.2)^b^	18.9 (14.2 to 28.4)	.000
BMI-Z score	−1.67 (−2.92 to 0.3)	−1.13 (−3.14 to 2.58)	−1.00 (−3.19 to 0.37)	.236	−1.28 (−3.19 to 2.58)^b^	−0.38 (−2.48 to 2.42)	.000

^a^Statistically significant compared with ND.

^b^Statistically significant compared with HC.

CD, celiac disease; ND, newly diagnosed; GFD, gluten-free diet compatible; non-GFD, non-gluten-free diet compatible; HC, healthy control; BMI-Z score, body mass index Z score.

**Table 2.** Mineral, BMD, and Fetuin-A Data of Study Groups

Variables	CD Group Mean ± SD or Med (Min-Max) (N = 59)					HC Group Mean ± SD or Med (Min-Max) (N = 29)	*P*
ND (N = 21)	GFD (N = 18)	Non-GFD (N = 20)	*P*	Total CD (N = 59)		
Ca (mg/dL)	9.48 ± 0.52	10.05 ± 0.27^a^	9.99 ± 0.30^a^	.005	9.88 ± 0.42	9.71 ± 1.48	.228
P (mg/dL)	5.01 (3.6 to 6.2)	4.52 (3.3 to 5.9)	4.60 (3.2 to 6.4)	.171	4.67 (3.2 to 6.4)^b^	4.00 (3.2 to 5.8)	.000
Mg (mg/dL)	2.00 (1.8 to 2.1)	1.90 (1.8 to 2.1)	1.85 (1.7 to 2.1)	.076	1.89 (1.7 to 2.1)^b^	2.15 (1.7 to 2.5)	.000
BMD Z-score	−0.97 ± 1.15	−0.51 ± 1.17	0.78 ± 1.18	.121	−0.76 ± 1.16	-	-
Fetuin A (µg/L)	130.5 (59.4 to 259.5)	146.9 (75.1 to 258.2)	134.4 (68.2 to 259.5)	.391	136.8 (59.4 to 259.5)^b^	112.9 (28.0 to 225.5)	.016

^a^Statistically significant compared with ND.

^b^Statistically significant compared with HC.

CD, celiac disease; ND, newly diagnosed; GFD, gluten-free diet compatible; non-GFD, non-gluten-free diet compatible; HC, healthy control; Ca, calcium; P, phospforus; Mg, magnesium; BMD, bone mineral density.

## References

[1] Tack GJ Verbeek WH Schreurs MW Mulder CJ . The spectrum of celiac disease: epidemiology, clinical aspects and treatment. Nat Rev Gastroenterol Hepatol. 2010;7(4):204 213. 10.1038/nrgastro.2010.23) 20212505

[2] Newton KP Singer SA . Celiac disease in children and adolescents: special considerations. Semin Immunopathol. 2012;34(4):479 496. 10.1007/s00281-012-0313-0) 22549889

[3] Phan CM Guglielmi G . Metabolic bone disease in patients with malabsorption. Semin Musculoskelet Radiol. 2016;20(4):369 375. 10.1055/s-0036-1592429) 27842429

[4] Kemppainen T Kröger H Janatuinen E et al.. Osteoporosis in adult patients with celiac disease. Bone. 1999;24(3):249 255. 10.1016/s8756-3282(98)00178-1) 10071918

[5] Sdepanian VL de Miranda Carvalho CN de Morais MB Colugnati FA Fagundes-Neto U . Bone mineral density of the lumbar spine in children and adolescents with celiac disease on gluten-free diet in Sao Paulo, Brasil. J Pediatr Gastroenterol Nutr. 2003;37(5):571 576. 10.1097/00005176-200311000-00013) 14581799

[6] Volkan B Fettah A İşlek A Kara SS Kurt N Çayır A . Bone mineral density and vitamin K status in children with celiac disease: is there a relation? Turk J Gastroenterol. 2018;29(2):215 220. 10.5152/tjg.2018.17451) 29749330 PMC6284705

[7] Mori K Emoto M Inaba M . Fetuin-A: a multifunctional protein. Recent Pat Endocr Metab Immune Drug Discov. 2011;5(2):124 146. 10.2174/187221411799015372) 22074587

[8] Termine JD Belcourt AB Conn KM Kleinman HK . Mineral and collagen-binding proteins of fetal calf bone. J Biol Chem. 1981;256(20):10403 10408. 10.1016/S0021-9258(19)68633-3) 6793579

[9] Wendel M Heinegård D Franzén A . A major non-collagenous 62 kDa protein from rat bone mineralized matrix is identical to pp63 a phosphorylated glycoprotein from liver. Matrix. 1993;13(4):331 339. 10.1016/s0934-8832(11)80029-6) 8412991

[10] Schafer C Heiss A Schwarz A et al. The serum protein alpha 2-Heremans-Schmid glycoprotein/fetuin-A is a systemically acting inhibitor of ectopic calcification. J Clin Invest. 2003;112(3):357 366. 10.1172/JCI17202) 12897203 PMC166290

[11] Westenfeld R Schäfer C Smeets R et al. Fetuin-A (AHSG) prevents extraosseous calcification induced by uraemia and phosphate challenge in mice. Nephrol Dial Transplant. 2007;22(6):1537 1546. 10.1093/ndt/gfm094) 17389622

[12] Westenfeld R Schäfer C Krüger T et al. Fetuin-A protects against atherosclerotic calcification in CKD. J Am Soc Nephrol. 2009;20(6):1264 1274. 10.1681/ASN.2008060572) 19389852 PMC2689898

[13] Schinke T Amendt C Trindl A Pöschke O Müller-Esterl W Jahnen-Dechent W . The serum protein alpha2-HS glycoprotein/fetuin inhibits apatite formation in vitro and in mineralizing calvaria cells. A possible role in mineralization and calcium homeostasis. J Biol Chem. 1996;271(34):20789 20796. 10.1074/jbc.271.34.20789) 8702833

[14] Price PA Toroian D Lim JE . Mineralization by inhibitor exclusion: the calcification of collagen with fetuin. J Biol Chem. 2009;284(25):17092 17101. 10.1074/jbc.M109.007013) 19414589 PMC2719347

[15] Toroian D Lim JE Price PA . The size exclusion characteristics of type I collagen: implications for the role of noncollagenous bone constituents in mineralization. J Biol Chem. 2007;282(31):22437 22447. 10.1074/jbc.M700591200) 17562713

[16] Ix JH Wassel CL Bauer DC et al. Fetuin-A and BMD in older persons: the Health Aging and Body Composition (Health ABC) study. J Bone Miner Res. 2009;24(3):514 521. 10.1359/jbmr.081017) 19016589 PMC2659522

[17] Chailurkit L Kruavit A Rajatanavin R Ongphiphadhanakul B . The relationship of fetuin-A and lactoferrin with bone mass in elderly women. Osteoporos Int. 2011;22(7):2159 2164. 10.1007/s00198-010-1439-3) 20963400

[18] Husby S Koletzko S Korponay-Szabó IR et al. European Society for Pediatric Gastroenterology, Hepatology, and Nutrition guidelines for the diagnosis of coeliac disease. J Pediatr Gastroenterol Nutr. 2012;54(1):136 160. 10.1097/MPG.0b013e31821a23d0) 22197856

[19] Neyzi O Gunoz H Furman A Bundak R Gokcay G Weight DF . height, head circumference and body mass index references for Turkish children. J Pediatr. 2008;51:1 14.

[20] Lewiecki EM Gordon CM Baim S et al. International Society for Clinical densitometry 2007 adult and pediatric official positions. Bone. 2008;43(6):1115 1121. 10.1016/j.bone.2008.08.106) 18793764

[21] Kalayci AG Kansu A Girgin N Kucuk O Aras G . Bone mineral density and importance of a gluten-free diet in patients with celiac disease in childhood. Pediatrics. 2001;108(5):E89. 10.1542/peds.108.5.e89) 11694673

[22] Blazina S Bratanic N Campa AS Blagus R Orel R . Bone mineral density and importance of strict gluten-free diet in children and adolescents with celiac disease. Bone. 2010;47(3):598 603. 10.1016/j.bone.2010.06.008) 20601293

[23] Binkert C Demetriou M Sukhu B Szweras M Tenenbaum HC Dennis JW . Regulation of osteogenesis by fetuin. J Biol Chem. 1999;274(40):28514 28520. 10.1074/jbc.274.40.28514) 10497215

[24] Heiss A DuChesne A Denecke B et al. Structural basis of calcification inhibition by alpha 2-HS glycoprotein/fetuin-A. Formation of colloidal calciprotein particles. J Biol Chem. 2003;278(15):13333 13341. 10.1074/jbc.M210868200) 12556469

[25] Herrmann M Schäfer C Heiss A et al. Clearance of fetuin-A--containing calciprotein particles is mediated by scavenger receptor-A. Circ Res. 2012;111(5):575 584. 10.1161/CIRCRESAHA.111.261479) 22753077

[26] Toroian D Price PA . The essential role of fetuin in the serum-induced calcification of collagen. Calcif Tissue Int. 2008;82(2):116 126. 10.1007/s00223-007-9085-2) 18097630

[27] Rasul S Ilhan A Reiter MH et al. Levels of fetuin-A relate to the levels of bone turnover biomarkers in male and female [patients with type 2 diabetes. Clin Endocrinol (Oxf). 2012;76(4):499 505. 10.1111/j.1365-2265.2011.04246.x) 21958193

[28] Ambroszkiewicz J Rowicka G Chelchowska M Gajewska J Strucińska M Laskowska-Klita T . Biochemical markers of bone metabolism in children with cow’s milk allergy. Arch Med Sci. 2014;10(6):1135 1141. 10.5114/aoms.2013.36906) 25624850 PMC4296058

[29] Özkan E Özkan H Bilgiç S et al. Serum fetuin-A levels in postmenopausal women with osteoporosis. Turk J Med Sci. 2014;44(6):985 988. 10.3906/sag-1308-28) 25552151

